# Association of musculoskeletal involvement with lung function and mortality in patients with idiopathic pulmonary fibrosis

**DOI:** 10.1186/s12931-024-02705-5

**Published:** 2024-02-08

**Authors:** Meenakshi Sridhar, Sandeep Bodduluri, Lanier O’Hare, Scott Blumhoff, Maria del Pilar Acosta Lara, Joao A. de Andrade, Young-Il Kim, Tracy Luckhardt, MerryLynn McDonald, Tejaswini Kulkarni

**Affiliations:** 1 Division of Pulmonary, Allergy and Critical Care Medicine, Department of Medicine, School of Medicine, University of Alabama at Birmingham, Birmingham, AL USA; 2 Lehigh Valley Health Network, Allentown, PA USA; 3 Division of Pulmonary, Allergy and Critical Care Medicine, Vanderbilt University, Nashville, TN USA; 4 Department of Genetics, School of Medicine, University of Alabama at Birmingham, Birmingham, AL USA; 5 Department of Epidemiology, University of Alabama at Birmingham, Birmingham, AL USA

**Keywords:** Frailty, Sarcopenia, Fat-free Mass index (FFMI), Interstitial lung disease, Pectoralis Muscle Area, Body mass index

## Abstract

Idiopathic pulmonary fibrosis (IPF) is a progressive disease associated with high mortality. Low muscle mass, frailty and sarcopenia lead to functional impairment that negatively impact quality of life and survival but are not used in clinical practice. We aimed to determine the association of Fat-free mass index (FFMI) and frailty with lung function, exercise tolerance and survival in patients with IPF. In this study, 70 patients with IPF underwent assessment of body composition, lung function, 6-min walk distance (6MWD) testing, hand grip strength, quality of life (QoL) assessment by St. George’s Respiratory questionnaire (SGRQ) and frailty assessment using the SHARE-FI tool. FFMI was calculated using pectoralis muscle cross-sectional area (PM-CSA) on CT chest images and the lowest quartile defined reduced muscle mass. Sarcopenia was defined as low FFMI and handgrip strength. Regression analyses were conducted to determine predictive value of frailty, low FFMI and sarcopenia on clinical outcomes. The Cox proportional hazards model was used to analyze the impact of FFMI and frailty score on survival. The mean age was 70 years with moderate impairment in lung function (mean ppFVC 68.5%, ppDLCO 45.6%). Baseline forced vital capacity (p < 0.001), diffusion capacity of lung for carbon monoxide (p =  < 0.01), 6WMD (p < 0.05) were significantly lower in frail patients compared to non-frail patients. BMI was found to closely correlate with FFMI (r = 0.79, p < 0.001), but not with frailty score (r = − 0.2, p = 0.07). Frailty was a significant predictor of FVC, DLCO, 6MWD, SGRQ scores when adjusted for age and gender. Muscle mass and sarcopenia were significant predictors of FVC, DLCO, but not 6MWD or QoL scores. Multivariate cox-proportional hazards ratio model adjusting for age and gender showed that frailty was significantly associated with increased mortality (HR = 2.6, 95% CI 1.1–6.1). Low FFMI (HR = 1.3, 95% CI 0.6–2.8), and sarcopenia (HR = 2.1, 95% CI 0.8–5.3), though associated with a trend to increased mortality, were not statistically significant. Frailty is associated with lower lung function and higher mortality in patients with IPF. Longitudinal evaluations are necessary to further determine the associations between low FFMI, sarcopenia and frailty with outcomes in IPF.

**Supplementary Information:**

The online version contains supplementary material available at 10.1186/s12931-024-02705-5.

## Introduction

Idiopathic pulmonary fibrosis (IPF) is a chronic lung disease with poor outcomes and limited therapeutic options [1]. IPF a disease of an aging population, where frailty and sarcopenia correlate with disability, hospitalizations, and mortality [2–4]. Sarcopenia is the loss of muscle mass and physical function, while frailty is a broader syndrome encompassing physical, social, cognitive, and psychological domains [2–5]. While low body mass index (BMI) has been associated with increased mortality and lower lung function in patients with interstitial lung disease [6, 7], loss of skeletal muscle mass may occur regardless of changes in BMI or weight, as is seen in sarcopenic obesity [8]. Fat-free mass index (FFMI) measurement offers a more sensitive tool than BMI to determine loss of skeletal muscle. FFMI correlates with Forced vital capacity (FVC), diffusion capacity of the lung for carbon monoxide (DLCO), 6-minute walk distance (6MWD), and survival [9]. FFMI correlates with skeletal muscle index as measured on CT chest imaging [10]. Low skeletal muscle area on CT, has been shown to be a strong predictor of mortality in IPF [10–13]. While prior studies of IPFs cohort have shown a correlation between radiographic measures of skeletal muscle mass and survival, they have not addressed the impact of frailty or functional impairment in this population [12, 13]. We aimed to close this gap by investigating the relationship of low CT derived FFMI, sarcopenia and frailty with lung function, quality of life, exercise tolerance and survival in patients with IPF.

## Methods

### Study population

Seventy patients with IPF were consecutively recruited from the ILD clinic from 2016 to 2018. Patients were either referred with an existing diagnosis or confirmed in clinic using latest ATS/ERS/JRS guidelines and multidisciplinary discussion (MDD). Time of diagnosis for this study was defined as the date of MDD at our institution. Medication history was obtained at the time of enrollment, including participation in clinical trials.

### Measurements

All patients underwent spirometry, lung volume, and diffusion capacity testing at the Pulmonary Function testing lab at the time of enrollment, and at 6 and 12 month follow up. The six-minute walk distance (6MWD) was measured by a trained respiratory therapist. The test is a self-paced test, along a flat course. St. George’s Respiratory Questionnaire (SGRQ) was administered that assesses three components—Symptoms, Activity, and impact on daily life. The Enrollment Measures of FVC, DLCO, 6MWD, SGRQ are considered the baseline value. The Survey of Health, Aging, and Retirement in Europe-Frailty Instrument (SHARE-FI) was administered to patients. The variables used to determine frailty are Exhaustion, Weight loss, Weakness as assessed by handgrip strength, Slowness, and Low activity. The SHARE-FI calculator is a free universally available tool that categorizes individuals into frail, pre-frail, and non-frail [14]. Muscle strength was assessed using a hand dynamometer. A cut-off of 30 kg for men and 20 kg for women was used to describe low handgrip strength [15].

**Fig. 1:**
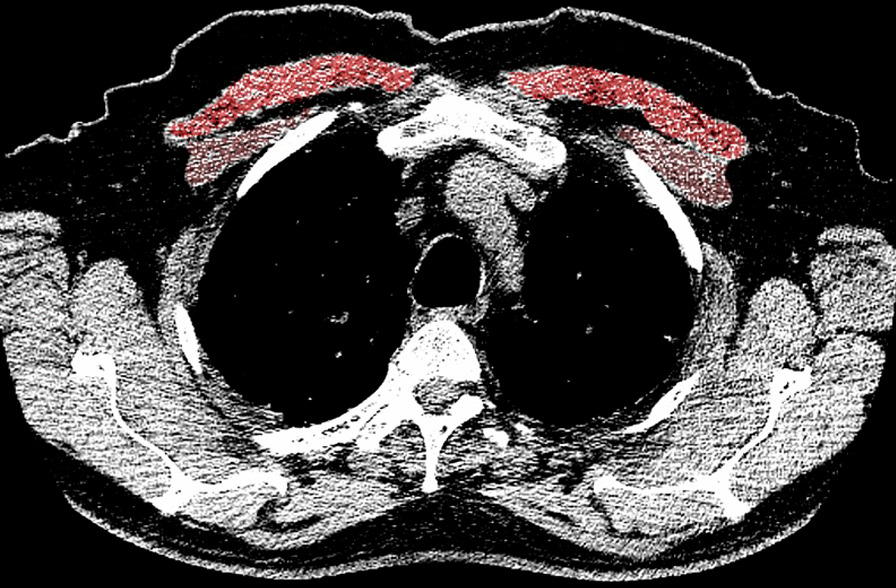
Measurement of pectoralis major and minor cross-section areas at T4 vertebra, using CT slicer

Muscle mass was measured using the CT chest image chronologically closest to the time of enrollment into study. A single axial image CT chest was used to determine the pectoralis major cross-sectional area (PM-CSA) at the level of the 4th Thoracic vertebrae (at the level of the aortic arch). Quantitative assessments of the pectoralis muscle area were done using CT Slicer 4.8 software. Muscles were manually shaded, using a predefined attenuation range of –50 and 90 HU, although this was modified on a case-by-case basis if the user finds excluded muscle regions. Measurement of the skeletal muscles was independently performed by two trained providers who are blinded to the clinical data (Fig. 1).

Fat-free mass from pectoralis muscle (FFM-PMA) and Fat free mass index were calculated from PM-CSA, height, weight, and gender of subjects using a formula that has been previously validated in a COPD cohort by McDonald et al., represented by the following equation[16].

$${\text{FFM - PMA = - 15}}{.9 + 0}{\text{.09}} \times {\text{PM - CSA (cm}}^{{2}} {) + 0}{\text{.35}} \times {\text{weight (kg) + 0}}{.2} \times {\text{height (cm) + 7}}{\text{.47 (if male)}}$$

$${\text{FFMI = FFM - PMA/height (m)}}^{{2}}$$

The lowest quartile of the fat-free mass index (FFMI) for each gender was used to define low FFMI. Sarcopenia was defined as the presence of low FFMI and low handgrip strength.

Survival time was the period from the date of study enrolment until death from the time of enrollment till December 2022. Whether the patients were still alive was determined by reviewing hospital records or publicly available obituaries. Exacerbations in the 12 months following enrollment were determined based on clinic and hospital records available.

### Exclusion criteria

IPF patients with evidence of airway obstruction or an FEV1/FVC of < 0.7, those unable to speak or comprehend the English language, listed for lung transplant, unable to use hands to measure grip strength, non-ambulatory for any reason, had evidence of sever or life-threatening organ damage were excluded from analyses. Severe or life-threatening organ damage exclusions included active malignancy or history of malignancy within 2 years, NYHA class III or IV congestive heart failure, liver cirrhosis with a MELD > 2, end-stage renal disease on dialysis and severe neurologic disease, i.e., Parkinson’s, Amyotrophic Lateral Sclerosis, or Dementia.

### Statistical analyses

Analyses were performed using R. Student’s t-test, Fisher, ANOVA, and Chi-square test were used for comparative analysis. Regression analyses were conducted to evaluate if BMI, low FFMI, frailty and sarcopenia predicted pulmonary function and exercise tolerance, and change in these parameters over 6 and 12 months adjusting for age and gender. Multivariate cox proportional hazards ratio was used to assess risk factors for mortality.

## Results

### Clinical characteristics

This study includes seventy patients with a diagnosis of IPF with 68.6% men and 31.4% women. The mean age was 70.4 years (SD = 6.9), the mean BMI of 29.4 (SD = 4.9). This cohort represented patients with moderately severe IPF, with mean FVC of 2.5L and ppFVC of 66.5% and mean DLCO of 11.2 cc/sec/mmHg and ppDLCO of 49.6%. Most patients were on antifibrotic therapy (68.5%) at the time of enrollment. Pirfenidone was the more commonly used agent (40%) compared to Nintedanib (28.5%). Antifibrotic use did not differ based on the presence of low FFMI (p = 1, Table 2), BMI category (p = 0.53, Table 3), sarcopenia (p = 0.45, Table 2) and frailty category (P = 0.55, Table 3). Only three patients were on steroids, for indications other than lung disease. Of the seventy patients, none were found to be underweight, 18.5% had a normal BMI, 40% were overweight and 41.4% were obese. Low FFMI was identified in 25.7% patients, and 12.8% had sarcopenia, the lowest quartile was used to define FFMI *(*≤ *18.47 in males and* ≤ *15.84 in females).* Based on the SHARE-frailty instrument, 40% were pre-frail, and 34.3% were frail. Charlson age comorbidity index (CACI) was elevated (> 4) in 70% patients, 48.5% had hypertension, 32.8% had a history of coronary artery disease and 71% had a previous history of smoking. There were sixteen exacerbations during the 1 year follow up, in fourteen patients of this cohort. The median time from diagnosis of IPF to enrollment was 1.44 years. The baseline characteristics of the total population are summarized in Table 1.

**Table 1:** Baseline characteristics of study population (n = 70)

**Demographics (mean, SD**)	
Age (yr.)	70.4 (6.95)
Males (n%)	68.5%
BMI (kg/m^2^)	29.4 (4.85)
Overweight (n%)	41.4%
Obese (n%)	40%
**Baseline pulmonary function tests (mean, SD)**	
FVC (in L)	2.5 (0.76)
FVC % predicted	66.5 (18.13)
DLCO (cc/sec/mmHg)	11.2 (4.47)
DLCO % predicted	49.6 (16.26)
**Comorbidities (n%)**	
Coronary artery disease	32.9%
Hypertension	48.5%
**Medications (n%)**	
Antifibrotics	68.5%
Steroids (> 10 mg/day prednisone equivalent)	4.3%
**Functional Status (mean, SD**)	
6MWD (ft)	991.4 (323.7)
SGRQ	46.3 (16.9)
Hand-grip strength (kg)	30.9 (10.8)
Low hand-grip strength (n%)	35.7%
**Frailty status**	
SHARE frailty score (mean, SD)	1.91 (1.58)
Frail (n%)	34.3%
Prefrail (n%)	40%
**Thoracic skeletal muscle mass (mean, SD)**	
Pectoralis muscle CSA (in cm^2^)	1010.8 (406.3)
FFMI	18.9 (1.9)
Low FFMI (n%)	25.7%
Sarcopenia (n%)	12.8%

BMI: Body Mass Index; FVC: Forced Vital Capacity; DLCO: Diffusion Capacity of Lung for Carbon Monoxide; 6MWD: Six-Minute Walk Distance; SGRQ: St. George's Respiratory Questionnaire; CSA: Cross sectional area; FFMI: Fat-Free Mass Index

### Comparative analysis of patients based on BMI, low FFMI, sarcopenia, and frailty

Patients with low FFMI had significantly lower BMI (23.9 vs 31.3 kg/m^2^, p < 0.001) and baseline DLCO (8.8 vs 12 cc/sec/mmHg, p = 0.01), a higher percentage of them were frail compared to those with normal FFMI. There were no significant differences in FVC, GAP index, comorbidities, 6MWD, oxygen requirement, at baseline among IPF participants with and without low FFMI. FFMI was found to correlate strongly with BMI (r = 0.79).

Sarcopenic patients had significantly lower baseline FVC (2.06 vs 2.58L, p = 0.01) and DLCO (6.5 vs 11.1 cc/sec/mmHg, p < 0.001) and ppDLCO (35.1 vs 51.7, p = 0.01), in addition to high GAP index BMI and CACI compared to those without sarcopenia. Sarcopenic patients also had a significantly lower BMI (22.9 vs 30.4 kg/m^2^, p < 0.001). Patients with high BMI had significantly higher baseline FVC, DLCO, hand grip strength and FFMI. Significantly fewer patients with high BMI (BMI > 24.9 kg/m^2^) were found to be frail compared to patients with normal BMI (26.3% vs 69.2%, p = 0.007). Frail patients had significantly lower lung function, 6MWD and quality of life compared to non-frail patients. Disease severity appears to worsen with increasing degree of frailty. Frail patients had significantly lower FFMI (18.2 v 19.5, p = 0.04) than non-frail patients. These findings suggest a functional reduction due to lower muscle mass, however, there was no difference in the BMI. Baseline oxygen requirement was higher in frail patients compared to prefrail and non-frail patients (2.8 vs 2.2 vs 0.6 L/min p = 0.003). Rate of exacerbation over a 12 month follow up period did not differ significantly based on FFMI (p = 0.4), Sarcopenia (p = 0.3), Frailty Category (p = 0.2), BMI (p = 0.2). There were no differences in the overall use of antifibrotic therapies based on the presence of musculoskeletal diseases. The results of the above analysis are summarized in Tables 2 and 3 and Additional file 1: Fig. S1.

**Table 2:** Comparison of baseline characteristics of patients with low FFMI and sarcopenia and those without

	FFMI		Sarcopenia	
	Normal FFMI (n = 52)	Low FFMI (n = 18)	Nonsarcopenic (n = 61)	Sarcopenic (n = 9)
Age	69.5	72.7	69.6	75.5
% Male	69.2%	66.7%	68.8%	66.7%
BMI	31.3***	23.9	30.4***	22.9
GAP Index	3.9	4.5	3.9	5.1
CACI > 4 (%)	65.4%	83.3%	65.5%*	100%
Antifibrotic therapy (%)	69.2%	66.7%	70.5%	55.5%
Baseline FVC (L)	2.57	2.35	2.58*	2.06
Baseline FVC (%)	66.81	65.72	67.3	61.2
Baseline DLCO (cc/sec/mmHg)	12*	8.8	11.9***	6.5
Baseline DLCO (%)	51.73	43.33	51.7*	35.1
6MWD (ft)	1003	958	1013	845
SGRQ score	45.91	47.34	46.17	47.01
Baseline Oxygen (%)	36.5%	50%	42.6%	22.2%
Median Baseline O2 requirement (L/min)	2	0	2	3
Frailty score	1.7	2.4	1.8	2.8
% Frail	25%*	61.1%	27.8%*	77.7%
Pectoralis muscle CSA (cm^2^)	1055.7	881.1	1031	871
Hand grip strength (kg)	31.9	27.7	32.35***	20.72
FFMI	19.6***	16.8	19.22***	16.52
IPF exacerbations in 12 months (n)	10	6	13	1

BMI: Body Mass Index; FVC: Forced Vital Capacity; DLCO: Diffusion Capacity of Lung for Carbon Monoxide; 6MWD: Six-Minute Walk Distance; SGRQ: St. George's Respiratory Questionnaire; FFMI: Fat-Free Mass Index. Significant differences are represented as follows, p < 0.05 = *, p < 0.01 = **, p < 0.001 = ***

**Table 3:** Comparison of baseline characteristics of patients based on frailty and BMI category

	Frailty category			BMI category	
	Non-frail (n = 18)	Pre-frail (n = 28)	Frail (n = 24)	Normal BMI (n = 13)	High BMI (n = 57)
Age	69.4	70	71.6	71.6	70.1
% Male	77.8%	67.8%	62.5%	61.5%***	70.2%
BMI	30.7	30	27.9	23.2***	30.8
GAP Index	3.3*	4	4.7*	5*	3.8
CACI > 4 (%)	55.5%	64.3%	87.2%	84.6%	66.7%
Antifibrotic use (%)	77.8%	67.8%	62.5%	61.5%	70.1%
Baseline FVC (L)	3***	2.5	2.1	1.9***	2.6
Baseline FVC (%)	76.4%*	66.1%	59.6%	57%	68.6%
Baseline DLCO (cc/sec/mmHg)	13.2**	11.7	9	7.4***	12
Baseline DLCO (%)	55.2%*	52%	42.5%	37.9**	52.2
Six-minute walk distance (ft)	1139*	981	893.2	978.2	994.5
SGRQ score	33.8***	45.3	56.8	52	44.9
Baseline Oxygen (%)	66.7%*	39.3%	20.8%	38.5%	40.3%
Median Baseline Oxygen requirement (L/min)	0	2	3	2	2
Frailty score	− 0.1***	1.8	3.6	2.6	1.7
% Frail	–	–	100%	69%**	26%
Pectoralis muscle CSA (cm^2^)	1098.9	1058.9	888.7	894.4	1037.4
Hand grip strength (kg)	34.3*	32.4	26.4*	25.5*	32.1
FFMI	19.5	19.1	18.2	16.5***	19.4
IPF exacerbations in 12 months (n)	5	3	8	6	10

BMI: Body Mass Index; FVC: Forced Vital Capacity; DLCO: Diffusion Capacity of Lung for Carbon Monoxide; SGRQ: St. George's Respiratory Questionnaire; FFMI: Fat-Free Mass Index. Significant differences are represented as follows, p < 0.05 = *, p < 0.01 = **, p < 0.001 = ***

BMI was found to correlate with FFMI (r = 0.79, p < 0.001), but not with hand grip-strength (r = 10.02, p = 0.9) or frailty score (r = − 0.2, p = 0.07). FFMI was found to have a statistically significant positive correlation with FVC (r = 0.35, p = 0.002), DLCO (r = 0.44, p < 0.001), ppDLCO (r = 0.25, p = 0.03), hand-grip strength showed a similar pattern of correlation. Frailty score had statistically significant negative correlation with FVC (r = − 0.34, p = 0.003), ppFVC (r = − 0.36, p = 0.002), DLCO (r = − 0.28, p = 0.02), ppDLCO (r = − 0.29, p = 0.01). BMI was only noted to have a correlation with DLCO (r = 0.33, p = 0.004). Additional file 1: Figs. S2 and S3 summarize these results.

In linear regression modeling (Table 4), low FFMI was a significant predictor of DLCO, and sarcopenia was a significant predictor of DLCO, and ppDLCO adjusted for sex and age. Frailty was a significant predictor of several traits including FVC, DLCO, 6MWD, quality of life and absolute change in FVC at 6 months.

**Table 4:** High BMI, low FFMI, frailty category and sarcopenia as predictors of pulmonary function adjusted for age and gender

	High BMI		Low FFMI		Prefrail		Frail		Sarcopenia	
	β	p-value	β	p-value	β	p-value	β	p-value	β	p-value
FVC	0.5	0.005	− 0.2	0.4	− 0.4	0.04	− 0.7	< 0.001	− 0.5	0.07
FVC%	13.2	0.01	− 2.5	0.6	− 11.9	0.02	− 19.9	< 0.001	− 9.1	0.16
DLCO	4.1	< 0.001	− 2.5	0.02	− 1.1	0.04	− 3.3	0.01	− 4.2	< 0.001
DLCO %	13.4	0.006	− 7.2	0.1	− 2.6	0.6	− 11.4	0.02	− 15.1	0.01
Six Minute walk distance	− 8.8	0.93	− 15	0.9	− 141.4	0.1	− 211.9	0.04	− 123.9	0.29
SGRQ	− 7.1	0.17	2.6	0.6	11.4	0.01	23.4	< 0.001	2.3	0.7
Absolute change in FVC% at 6 months	− 0.1	0.61	3.1	0.3	− 2.7	0.4	− 7.1	0.04	2.8	0.49
Absolute Change in FVC% at 12 months	2.5	0.05	2.4	0.4	− 0.1	1	1.3	0.65	− 2	0.61

BMI: Body Mass Index; FVC: Forced Vital Capacity; DLCO: Diffusion Capacity of Lung for Carbon Monoxide; SGRQ: St. George's Respiratory Questionnaire; FFMI: Fat-Free Mass Index. β is the degree of change in outcome variable for presence of high BMI, low FFMI, Frailty, Sarcopenia

Male gender was found to be a significant predictor of FVC, ppFVC, and DLCO but not of ppDLCO, baseline 6MWD or SGRQ score, change in FVC at 6 months or 12 months on univariate analysis. This remained significant when adjusted for age, high BMI, low FFMI, sarcopenia and frailty category individually.

### Survival analysis

**Fig. 2:**
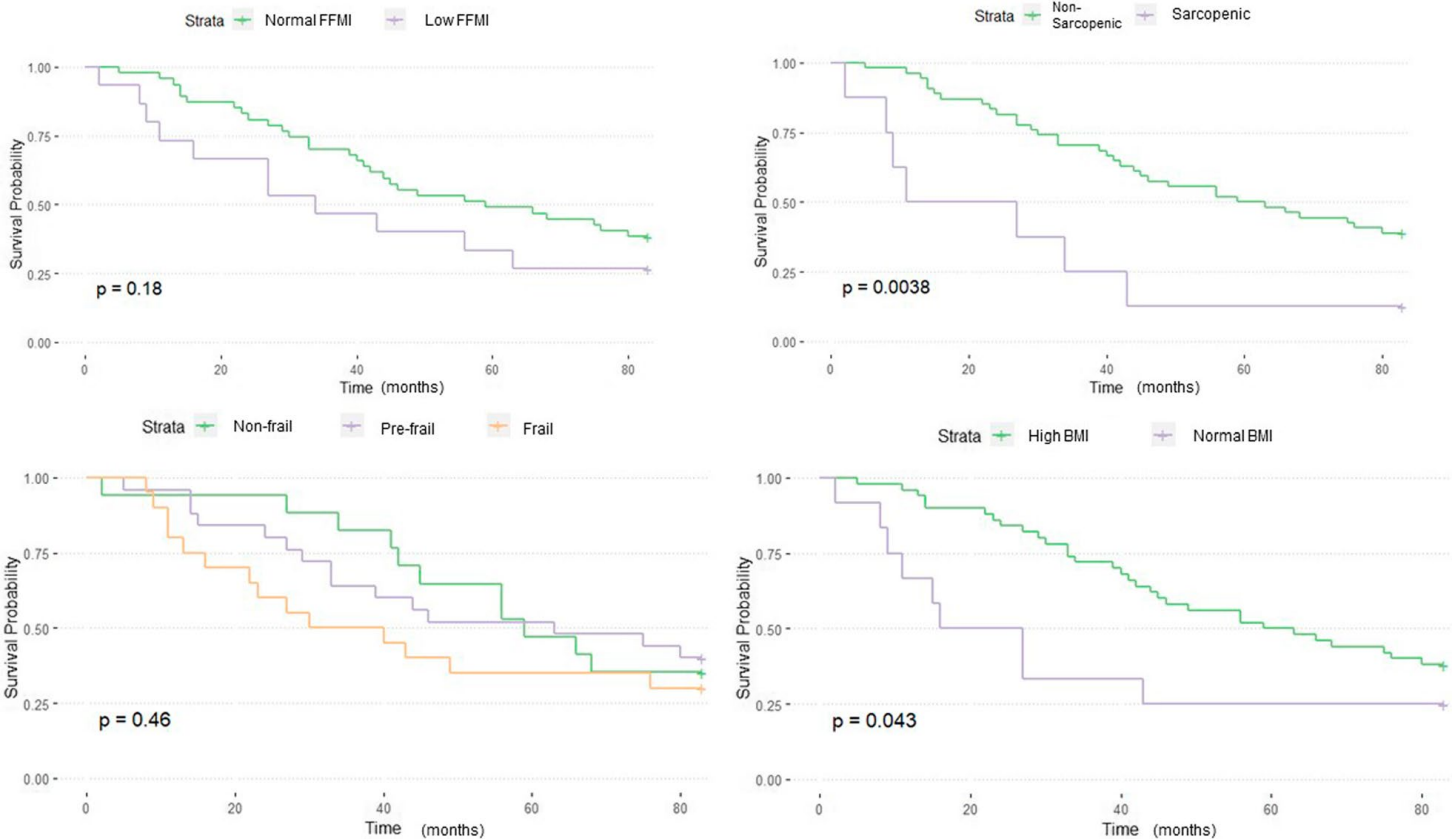
Kaplan–Meier survival curve for IPF patients based on FFMI, sarcopenia, frailty category and BMI

**Fig. 3:**
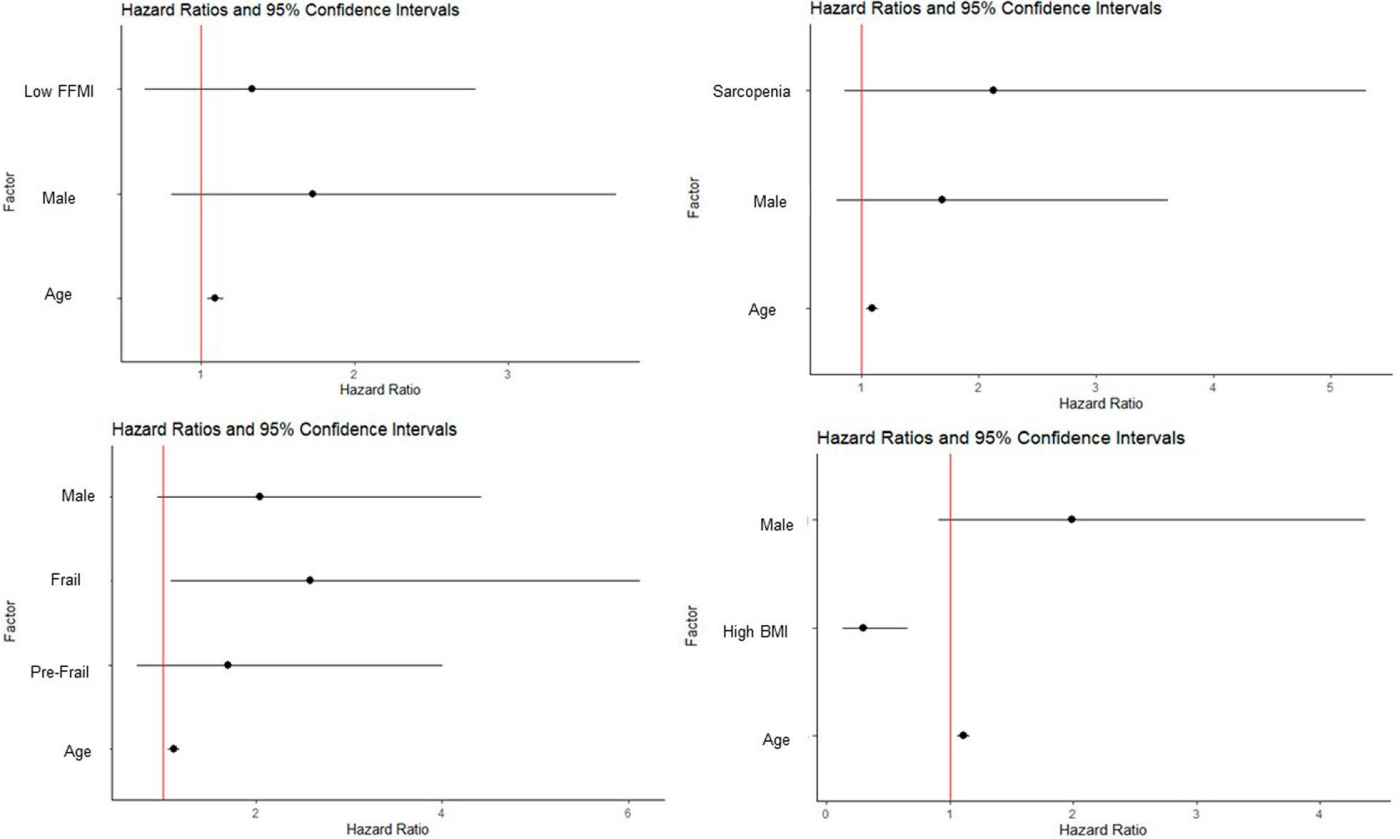
Musculoskeletal comorbidities as predictors of mortality. Forest plot representing Cox-proportional hazards ratios

Kaplan–Meier survival curves showed significantly lower median survival in patients with sarcopenia (19 vs 61 months, p = 0.004) and significantly higher in those with higher BMI (61 vs 21.5 months, p = 0.04), however, frailty and low FFMI did not have a similar effect. Figure 2 summarizes the results of this analysis. Multivariate Cox-proportional Hazards ratio model adjusting for age and gender indicated low FFMI (HR = 1.3, 95% CI 0.6–2.8), and sarcopenia (HR = 2.1, 95% CI 0.8–5.3) were associated with a trend to increased mortality, these were not statistically significant. Frailty showed a significant association with increased risk of mortality (HR = 2.6, 95% CI 1.1–6.1) and high BMI was shown to have reduced risk (HR = 0.3, 95% CI 0.14–0.66). Summarized in Fig. 3. Frailty continued to be associated with higher risk of mortality with the addition of baseline FVC (HR = 1.7, 95% CI 0.6–4.8), DLCO (HR = 1.9, 95% CI 0.8–4.8) and baseline oxygen requirement (HR = 2.3, 95% CI 0.96–5.9) to the above model individually, however this was no longer statistically significant (Additional file 1: Fig. S4).

## Discussion

We compared the relationship of BMI, low FFMI, sarcopenia and frailty with pulmonary, extrapulmonary outcomes and survival in patients with IPF. Although low FFMI and sarcopenia had some overlapping criteria with frailty, we found frail patients were more likely to experience adverse pulmonary and extrapulmonary outcomes than those characterized by low FFMI or sarcopenia. SHARE-FI includes fatigue, loss of appetite, physical activity, functional difficulties, and grip strength. These components of the frailty score may be more relevant than low FFMI and grip strength alone, suggesting that global loss of function captured by frailty assessment is a key harbinger of decline.

The results of the above regression analyses suggested that other components of the frailty score may play a bigger role in determining lung function compared to only hand grip strength, which was also used to define sarcopenia. Frailty has shown to be associated with increased hospitalizations, time to discharge and worse quality of life in patients with fibrotic ILDs [17]. We found that frail patients had significantly lower baseline lung function, exercise tolerance and quality of life compared to non-frail patients. This is similar to a previous smaller study [18] where frailty was associated with lower lung function and 6WMD and higher symptom burden, while pectoralis muscle area did not approach significance in older IPF patients [18]. Our study additionally showed that frailty was a significant predictor of change in FVC at 6 months and mortality. On adjusting for baseline lung function frailty was still associated with higher risk of mortality in this cohort, though this was no longer statistically significant, a larger study is needed to determine the true impact of frailty on mortality in IPF. While the cause of death was not addressed in this study, others have shown acute exacerbations and respiratory failure are the most common cause of mortality [19, 20]. Physical activity is a key component of frailty scores that have been associated with lower lung function and exercise tolerance in chronic lung disease [21]. Measures of physical performance, like gait speed allow better discrimination of mortality risk [22]. This may explain how the additional components of SHARE-FI like functional difficulties, and physical activity contribute to exercise tolerance and quality of life compared to hand-grip strength, which was used to define sarcopenia here.

In this study, we measured FFMI using PM-CSA on routine CT imaging and a formula previously validated in a COPD cohort [16]. Low FFMI was a significant predictor of DLCO, this result was similar to the findings of a Canadian cohort of ILD patients [17, 23, 24]. FFMI, frailty score and handgrip strength were found to have a weak but statistically significant positive correlation with FVC, ppDLCO and DLCO, while BMI only showed a weak correlation with DLCO, this may indicate that thoracic muscle mass and muscle strength have a higher influence on spirometric measurements. Median survival was significantly lower in sarcopenic patients. Lower FFMI and higher frailty scores are associated with a greater risk of mortality in our cohort, independent of age and sex.

BMI was shown to correlate significantly with FFMI which may be due to the inclusion of weight and height in the calculation of FFMI [16], but not frailty scores or grip strength. A challenge with using low BMI only to understand musculoskeletal diseases is even overweight and obese pulmonary patients can have low FFMI [25]. In fact, in the current cohort of IPF patients no individuals had low BMI despite many meeting criteria for frailty and/or sarcopenia. This underscores the importance of investigating indicators other than BMI, that influence lung function and patient-centered outcomes in IPF*.* Male gender and age conferred increased risk of mortality in this IPF cohort, which is similar to prior studies [1, 26]. Here, male gender was found to be a significant predictor of FVC, ppFVC, and DLCO and remained significant when adjusted for age, high BMI, low FFMI, sarcopenia and frailty category individually. The rate of exacerbation over a 12-month period did not differ significantly based on Frailty, FFMI, sarcopenia or BMI status. Most patients in this cohort were on antifibrotic therapy (68.5%) and its use was similar across all groups.

While this was a small single-center cohort, differences between BMI, FFMI, frailty and sarcopenia in IPF patients are apparent. We are limited by the lack of a universally accepted definition of sarcopenia; we used a combination of low muscle mass and hand grip strength, but this requires validation. The formula used to calculate FFMI was derived from a COPD cohort, and the lowest quartile of FFMI was used to define low muscle mass like other studies in ILD cohorts [10–12]. This, however, may not adequately represent the burden of disease. Finally, the frailty score utilized here may identify functional decline but may not differentiate individual factors that infer biological correlation and predict poor outcomes.

## Conclusions

Frailty is a syndrome associated with multidimensional loss of function and our study shows its impact on outcomes in IPF. In this cohort frailty was found to have significant association with poor lung function and survival, when adjusted for age and gender. Low FFMI and sarcopenia showed a similar trend but were not statistically significant. BMI was found to closely correlate with FFMI but not with frailty score, suggesting that functional impairment may not be fully captured by BMI, and there is a role for additional parameters to evaluate the effects of musculoskeletal dysfunction in IPF. Further investigation is also needed into their role in non-IPF ILDs.

### Supplementary Information

**Additional file 1: Figure S1.** Relationship of FVC and DLCO with Fat-Free mass index and Frailty category. **Figure S2.** Correlation of FVC and Musculoskeletal Comorbidities. **Figure S3.** Correlation of DLCO and Musculoskeletal Comorbidities. **Figure S4.** Frailty as a predictor of mortality adjusting for baseline disease severity. Forest plot representing Cox-proportional hazards ratios. **Table S1.** Comparison of FFMI and BMI as predictors of lung function.

## Data Availability

All data was stored in an encrypted password protects file, that did not include patient identifiers. Any additional data can be requested from the authors (Meenakshi Sridhar and Tejaswini Kulkarni), if not already included in the manuscript.
