# Supplementary material for: Association of musculoskeletal involvement with lung function and mortality in patients with idiopathic pulmonary fibrosis
Source: Respir Res. 2024 Feb 8;25:81. doi: 10.1186/s12931-024-02705-5 (PMC10851557; doi:10.1186/s12931-024-02705-5)

Table S5 – Comparison of FFMI and BMI as predictors of lung function

|  | BMI | FFMI | High BMI | Low FFMI |
| --- | --- | --- | --- | --- |
|  | R^2^ | R^2^ | R^2^ | R^2^ |
| FVC | 0.22 | 0.2 | 0.2 | 0.26 |
| FVC% | 0.12 | 0.11 | 0.11 | 0.17 |
| DLCO | 0.33 | 0.31 | 0.31 | 0.35 |
| DLCO% | 0.09 | 0.08 | 0.08 | 0.15 |
| *FVC - Forced Vital Capacity, DLCO- Diffusion Capacity of Lung for Carbon Monoxide, FFMI - Fat-Free Mass Index, BMI- Body mass index* | | | | |

*Figure S1- Relationship of FVC and DLCO with Fat-Free mass index and Frailty category.*

Figure S2 - Correlation of FVC and Musculoskeletal Comorbidities.

Figure S3 – Correlation of DLCO and Musculoskeletal Comorbidities.

*Figure S4- Frailty as a predictor of mortality adjusting for baseline disease severity. Forest plot representing Cox-proportional hazards ratios.*

*Figure S1- Relationship of FVC and DLCO with Fat-Free mass index and Frailty category.*


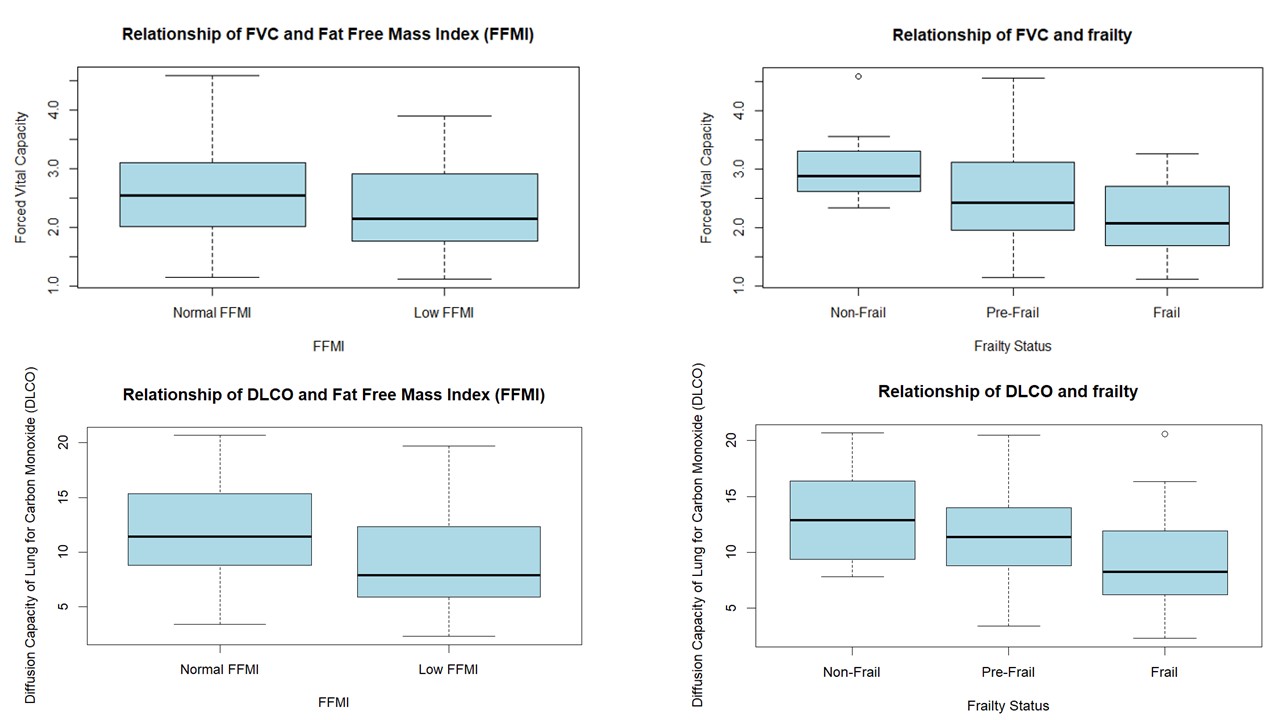


Figure S2 - Correlation of FVC and Musculoskeletal Comorbidities.


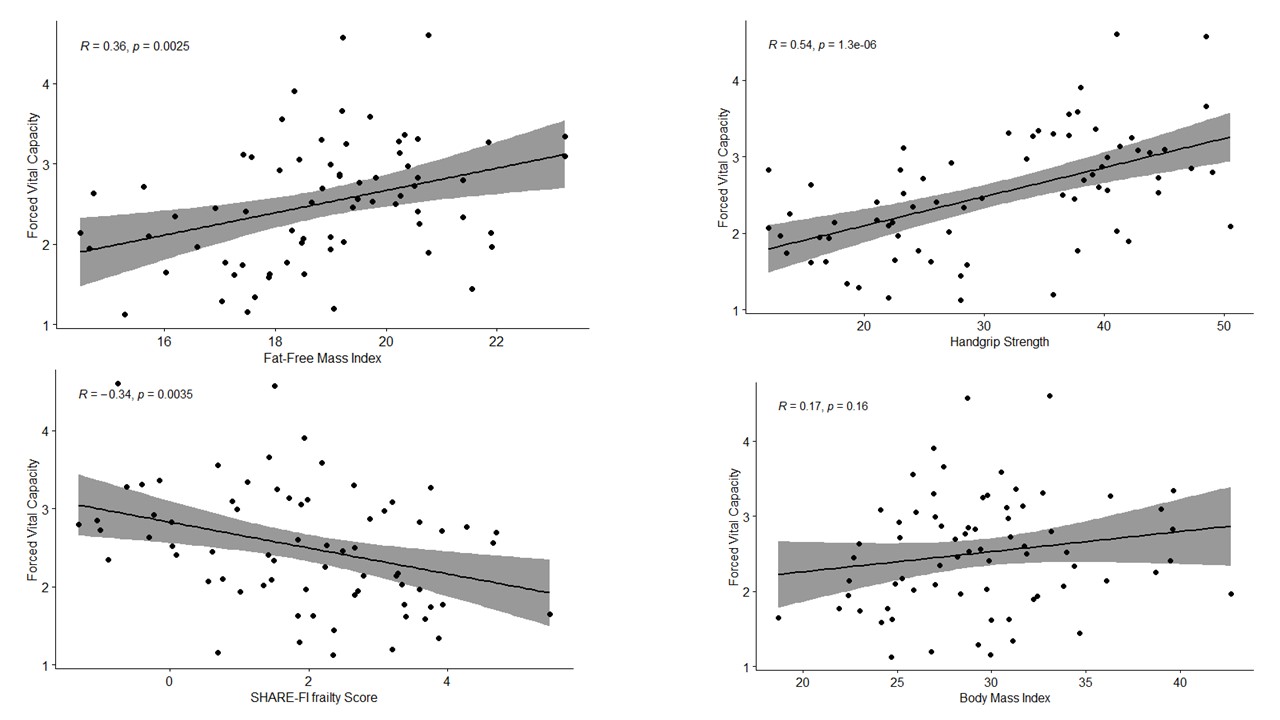


Figure S3 – Correlation of DLCO and Musculoskeletal Comorbidities.


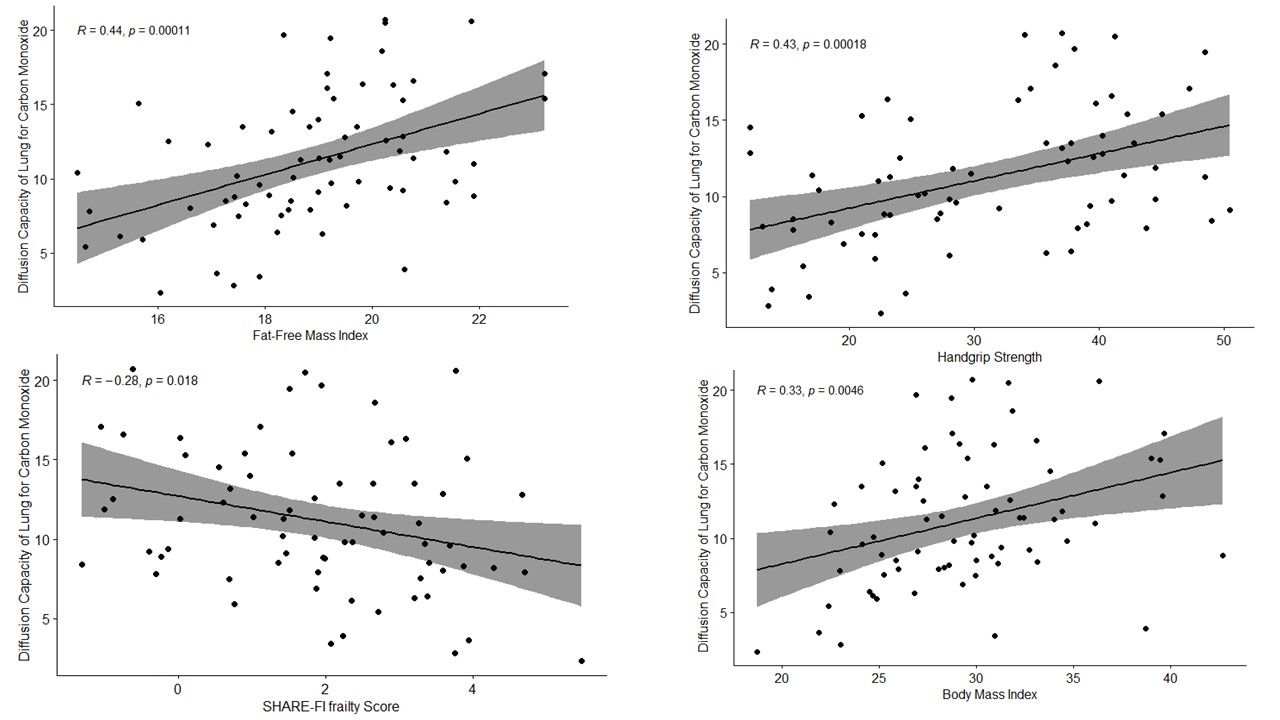


*Figure S4- Frailty as a predictor of mortality adjusting for baseline disease severity. Forest plot representing Cox-proportional hazards ratios.*


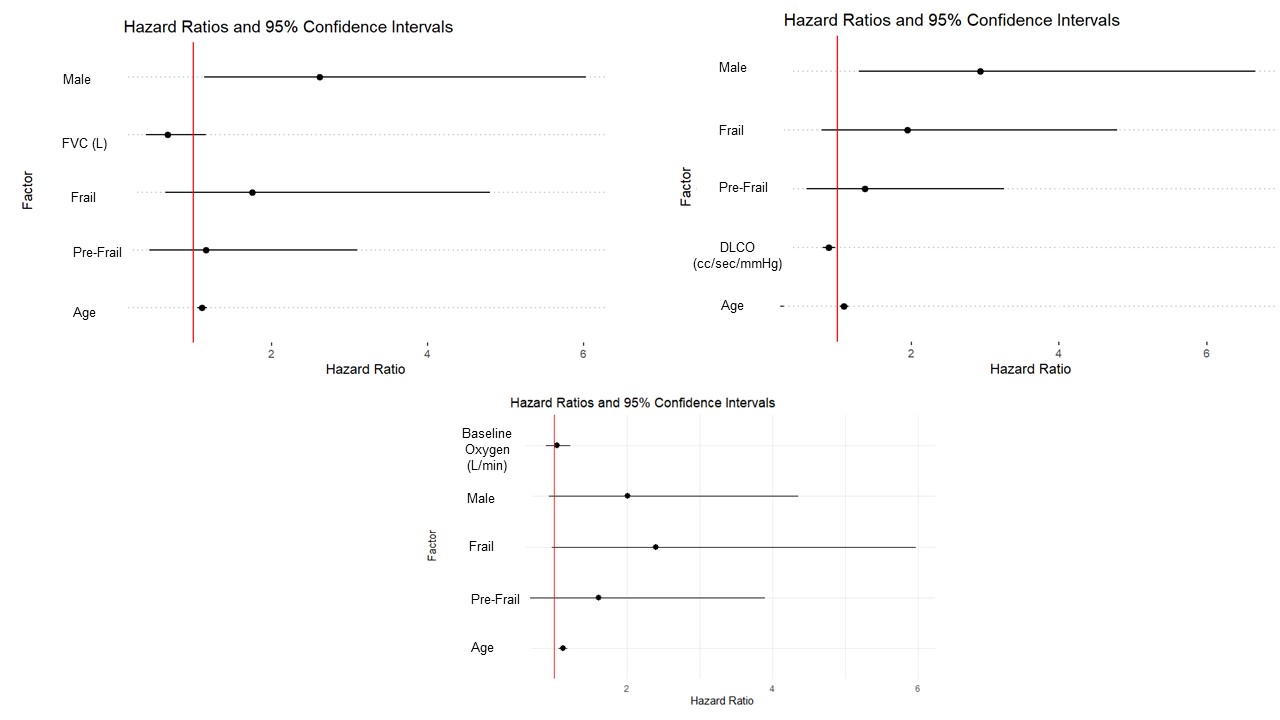

Supplement: Supplementary file 1 — Additional file 1: Figure S1. Relationship of FVC and DLCO with Fat-Free mass index and Frailty category. Figure S2. Correlation of FVC and Musculoskeletal Comorbidities. Figure S3. Correlation of DLCO and Musculoskeletal Comorbidities. Figure S4. Frailty as a predictor of mortality adjusting for baseline disease severity. Forest plot representing Cox-proportional hazards ratios. Table S1. Comparison of FFMI and BMI as predictors of lung function. [file 12931_2024_2705_MOESM1_ESM.docx]
